# Metacognition and cognitive dysfunction in post-COVID condition

**DOI:** 10.3389/fpsyg.2026.1786395

**Published:** 2026-03-09

**Authors:** Silvia Oliver-Mas, Cristina Delgado-Alonso, María Díez-Cirarda, Eleonora Catricalà, Constanza Cuevas, María Valles-Salgado, Yadhira Barroso, Juan Ignacio López-Carbonero, José Manuel Alcalá Ramírez del Puerto, Stefano F. Cappa, Jordi A. Matias-Guiu

**Affiliations:** 1 Department of Neurology, Hospital Clínico San Carlos, San Carlos Health Research Institute (IdISSC), Universidad Complutense de Madrid, Madrid, Spain; 2 ICON Cognitive Neuroscience Center, University Institute for Advanced Studies IUSS, Pavia, Italy

**Keywords:** cognitive impairment, COVID-19, metacognition, neuropsychological, post-covid condition

## Abstract

**Background:**

The mechanisms associated with cognitive issues in post-COVID condition (PCC) are still under debate. Metacognition refers to the ability to reflect and evaluate one’s cognitive functioning and remains unexplored in this condition. This study aimed to investigate both local and global metacognition in individuals with PCC according to the presence of objective cognitive impairment and to assess the relationship between metacognitive abilities and fatigue, depression, and anxiety.

**Methods:**

A total of 74 PCC (mean age = 51.45 ± 8.74 years; 78.4% female) patients and 49 healthy controls (HC) (mean age = 49.55 ± 8.84 years; 85.7% female) were included in this cross-sectional study. All participants completed a comprehensive neuropsychological battery. Local metacognition was assessed through task-specific performance estimates, collected through predictions and postdictions of the performance in the neuropsychological assessment. Global metacognition was assessed via two self-report instruments. PCC patients were classified as cognitively preserved (PCC-CP = 43) and cognitively impaired (PCC-CI = 31).

**Results:**

PCC patients showed reduced accuracy in both local and global metacognition compared to HC. Regarding local metacognition, PCC-CI patients significantly overestimated their performance in attention, executive function and memory. For global metacognition, both PCC-CP and PCC-CI underestimated their global cognitive abilities compared to HC. Global metacognitive scores were negatively correlated with fatigue and depression only in the PCC-CP group.

**Conclusion:**

PCC exhibit impaired local and global metacognitive accuracy, with differences according to the presence of objective cognitive impairment. These findings underscore the importance of assessing cognitive performance and metacognitive abilities in PCC patients to better understand subjective cognitive complaints and inform targeted rehabilitation strategies.

## Introduction

Post-COVID condition (PCC) is defined by the presence of new or persistent symptoms that persist at least 3 months after a probable or confirmed SARS-Cov-2 infection, last for at least 2 months, and cannot be explained by an alternative diagnosis (Soriano et al., 2022). Common symptoms include fatigue, cognitive dysfunction, shortness of breath, sleep disturbances, and mood symptoms, which significantly impact daily functioning and occupational status (Delgado-Alonso et al., 2022; Peter et al., 2022) and produce a high burden on healthcare systems (Mirin, 2022).

Cognitive symptoms are among the most frequently reported in individuals with PCC (Florencio et al., 2022; Premraj et al., 2022). Neuropsychological assessments have shown that attention, processing speed, episodic memory and executive functions are the most frequently impaired cognitive functions in PCC patients with cognitive complaints. Several studies have related cognitive dysfunction to functional and structural brain changes in patients with PCC (Hosp et al., 2024; Douaud et al., 2022; Díez-Cirarda et al., 2023; Díez-Cirarda et al., 2023). Other studies, however, have emphasized the possible impact of neuropsychiatric disorders or other associated factors (e.g., fatigue, sleep problems) (Delgado-Alonso et al., 2023) in the causation of cognitive issues (Verveen et al., 2024; Koterba et al., 2025). In this regard, the term of “brain fog” has been used in PCC literature and other medical disorders to define subjective cognitive symptoms that could implicate objective cognitive deficits, fatigue, and affect (Denno et al., 2025). Overall, the controversy about PCC and the pathophysiology of cognitive symptoms is an open debate, and some authors have suggested a potential link between PCC and functional neurological disorders (Alonso-Canovas et al., 2023).

Although there is a growing recognition of cognitive dysfunction in PCC patients, little is known about how PCC patients perceive and evaluate their own cognitive performance. Metacognition broadly refers to the ability to reflect and evaluate one’s cognitive functioning (Flavell, 1979) and can be divided into two components: local metacognition, which refers to one’s task-specific judgments and is typically assessed by asking individuals to estimate their performance before and after completing a task (metacognitive knowledge and metacognitive experience, respectively); and global metacognition, which involves broader self-assessments of general cognitive abilities, often measured through questionnaires or subjective memory ratings (Bacon et al., 2011; Van Camp et al., 2019; Torres et al., 2016). Evaluating metacognition is important because altered metacognitive beliefs or awareness can impact behaviour, decision-making and quality of life (Al-Aloucy et al., 2011; Yeo et al., 2021). In this regard, metacognition plays an important role in activities of daily living, as it supports the ability to manage complex tasks. Therefore, impairments in metacognition may be linked to reduced functional independence in daily life activities (Davies et al., 2017; Lamash, 2025).

Previous neuroimaging studies suggest that metacognitive impairments are associated with brain changes. For instance, in patients with mild amnestic cognitive impairment, metacognitive avoidance strategies have been positively correlated with left and right amygdala and parahippocampal volumes (Giannouli and Tsolaki, 2023). In addition, in functional cognitive disorder, metacognition appears to be impaired, leading to mismatches between subjective cognitive complaints and objective performance, suggesting an underestimation of actual cognitive abilities (Bhome et al., 2022; Cappa et al., 2024).

Despite its importance, the role of metacognition in PCC remains largely unexplored. Understanding how PCC affects metacognitive functioning could guide more effective interventions (Cabreira et al., 2023), help to validate patients’ experiences and inform clinical care. Therefore, the present study aimed to investigate both local and global metacognition in individuals with PCC, according to the presence of objective cognitive impairment and to assess the relationship between metacognitive abilities and fatigue, depression, and anxiety.

## Methods

### Participants and study design

We conducted a cross-sectional study involving 123 participants: 49 healthy controls (HC) and 74 PCC. This study represents a follow-up assessment of a cohort of patients previously examined (Díez-Cirarda et al., 2023; Delgado-Alonso et al., 2022). Patients were examined at least 2 years after the first neuropsychological examination, approximately 52 ± 5.62 months after the acute SARS-CoV-2 infection.

All the participants in the study were native Spanish speakers. Inclusion criteria for PCC patients were: (1) Previous history of COVID-19 with positive RT-PCR; (2) Diagnosis of PCC according to WHO criteria (Soriano et al., 2022); (3) Cognitive complaints temporally related to the SARS-CoV-2 infection. Exclusion criteria were the following: (1) The existence of cognitive dysfunction or cognitive complaints before SARS-CoV-2 infection; (2) Previous or current diagnosis of neurological disorder (e.g., stroke or brain injury), psychiatric disease (e.g., schizophrenia) or infection in the central nervous system potentially associated with cognitive impairment; (3) History of substance abuse; (4) Sensory or motor deficits that may hinder the neuropsychological assessment. For the HC group, exclusion criteria were: (1) subjective cognitive complaints or cognitive impairment; (2) medical history of neurological disorders associated with cognitive impairment (e.g., traumatic brain injury); (3) present or previous psychiatric disorder with a potential impact on cognitive functions; (4) History of substance abuse; (5) Cognitive impairment due to uncontrolled medical conditions or drugs at the moment of the assessment; (6) Sensory disorder which can affect the results of cognitive assessment.

The study was conducted at the Department of Neurology of Hospital Clinico San Carlos, in Madrid. Participants were recruited from referrals to a specific consultation for post-COVID patients in the same department.

### Neuropsychological assessment

For patients with PCC, the neuropsychological protocol consisted of a standardized battery that was administered in person by a trained neuropsychologist. The assessment included: forward and backwards digit span, Corsi block-tapping test, Symbol Digit Modalities Test, Boston Naming Test, Judgment Line Orientation, Rey-Osterrieth Complex Figure (copy and recall at 3, 30 min, and recognition), Free and Cued Selective Reminding Test, verbal fluencies (animals in 1 min and words beginning with “p”), Stroop Color- Word Interference Test (Stroop W: reading; Stroop C; color naming; Stroop WC: interference), and the Visual Object and Space Perception Battery (object decision, progressive silhouette, number location, position discrimination). The HC group underwent the same protocol, except Corsi test, Boston Naming Test, delayed recall and recognition of the Rey-Osterrieth Complex Figure, and two Visual Object and Space Perception Battery subtests (object decision, progressive silhouette). Raw scores were adjusted by age, education and sex (when necessary) according to the normative data available in our country (Peña-Casanova et al., 2009; Peña-Casanova et al., 2012).

The neuropsychological tests were selected to assess the cognitive domains most commonly affected in PCC patients according to the IC-CoDi-COVID taxonomy (Matias-Guiu et al., 2023) and including attention/processing speed, executive function, memory, language, and visuospatial abilities. All tests administered have normative data available for the Spanish population (Peña-Casanova et al., 2012; Peña-Casanova et al., 2009).

Patients were classified as cognitively preserved (PCC-CP) or cognitively impaired (PCC-CI) according to the IC-CoDi-COVID criteria, a previously developed methodology for the diagnosis of cognitive impairment associated with COVID-19 (Matias-Guiu et al., 2023). This approach is based on the empirically driven taxonomy developed for epilepsy after the consensus between the International League Against Epilepsy (ILAE) and the International Neuropsychological Society (IC-CoDE) (Norman et al., 2021). The following cognitive domains and test scores were included in this classification system: (a) attention/processing speed (Symbol Digit Modalities Test, Stroop W); (b) executive function (Stroop WC; Digit span backwards); (c) episodic memory (Free and Cued Selective Reminding Test-total delayed recall and Rey-Osterrieth Complex Figure-memory at 3 min); (d) Visuospatial function (Visual Object and Space Perception Battery-number location and Judgment Line Orientation); (e) Language (semantic fluency and Boston Naming Test). For this study, we used the −1.0 SD cutoff, considering a cognitive domain as impaired when two tests of the same domain are below the −1.0 SD cutoff.

Additionally, we estimate the global cognitive performance as the mean of the z-score of the 10 neuropsychological test scores included in the criteria, covering the 5 cognitive domains.

### Metacognition assessment

To assess local metacognition, participants were asked to estimate their expected performance immediately before (prediction) and after (postdiction) completing each task. They responded using a Likert type scale, both before (prediction) and after (postdiction) completing each test. Before the test, the prompt was: “Compared to healthy people of my age, I believe that my performance on the upcoming test will be: (−3) profoundly below average, (−2) well below average, (−1) below average, (0) average, (1) above average, (2) well above average, and (3) superior.” After completing the test, the prompt was: “Compared to healthy people of my age, I believe that my performance on this test was: (−3) profoundly below average, (−2) well below average, (−1) below average, (0) average, (1) above average, (2) well above average, and (3) superior. This procedure was applied to the following test: digit span backwards, Stroop (part W and WC), Symbol Digit Modalities Test, Free and Cued Selective Reminding Test total delayed recall, Rey-Osterrieth Complex Figure memory at 3 min, Judgment Line Orientation, Visual Object and Space Perception Battery (number location) and semantic fluency.

For the assessment of global metacognition, we administered the Multifactorial Memory Questionnaire (MMQ) (Troyer and Rich, 2002). This scale was designed to assess metamemory. Items are rated on a 5-point Likert scale. It comprises three subscales: (1) MMQ-Satisfaction, which measures an individual’s perceived satisfaction and concerns regarding their memory performance. It includes 18 items, with scores ranging from 0 to 72, where higher scores indicate greater satisfaction; (2) MMQ-Ability, which evaluates self-perceived memory performance in daily life. This subscale consists of 20 items and scores range from 0 to 80, where higher scores reflect better self-reported memory ability; (3) MMQ-Strategy, which evaluates whether individuals employ practical memory strategies and external aids in their daily lives. This subscale includes 19 items with scores ranging from 0 to 76, with higher scores indicating more frequent use of memory strategies.

We also administered Meta-Cognition Questionnaire-30 (MCQ-30) for global metacognition (Ramos-Cejudo et al., 2013). This questionnaire assesses metacognitive beliefs through 30 items across five subscales: “Positive beliefs about worry”; “Negative beliefs of uncontrollability and danger”; “Cognitive confidence”; “Need to control thoughts”; and “Cognitive self-consciousness.” Items are rated on a 4-point Likert scale with total scores ranging from 30 to 120 points. Higher scores reflect greater conviction of maladaptive metacognitive beliefs.

### Clinical and neuropsychiatric assessments

For the clinical assessment of patients with PCC, the following self-report instruments were administered: the Beck Depression Inventory-II (Beck et al., 1996), Modified Fatigue Impact Scale (MFIS), which includes a total score MFIS-Total, and three subscales (MFIS-Physical, MFIS-Cognitive, MFIS-Psychosocial) (Kos et al., 2005), Pittsburgh Sleep Quality Index (Buysse et al., 1989), State–Trait Anxiety Inventory (Spielberger et al., 1983). The following clinical cutoff scores, based on previous studies, were applied: State–Trait Anxiety Inventory ≥40 indicated clinically significant anxiety; Beck Depression Inventory-II ≥ 19 reflected moderate or severe depression (Beck and Carbin, 1988), Pittsburgh Sleep Quality Index *>*5 denoted poor sleep quality (Buysse et al., 1989), and MFIS ≥38 was indicative of clinically relevant fatigue (Strober et al., 2020).

The FLEI questionnaire was also administered to assess subjective mental ability. It consists of 35 questions about subjective cognitive complaints associated with attention (FLEI-Attention), executive functions (FLEI-Executive Functions) and memory (FLEI-Memory). A total score (FLEI-Total) is also obtained as the sum of FLEI-Memory, FLEI-Attention and FLEI-Executive Function (Beblo et al., 2011).

### Metacognitive indices

Age- and education-adjusted scaled scores from the neuropsychological tests were converted to the same scale used in the prediction and postdiction (from −3 to 3) according to the z-scores. After that, we calculated two local metacognitive indices: (i) Metacognitive knowledge difference score (MKDS): patient prediction minus actual performance; (ii) Metacognitive experience difference score (MEDS): patient postdiction minus actual performance. Accordingly, a score of 0 reflects perfect metacognitive accuracy, meaning that the subjective estimation matches the actual performance. Higher and lower scores are interpreted, respectively, as overestimation and underestimation of the own cognitive performance, if differences in the indices against healthy controls were significant.

For global metacognition, we converted into z-scores the MMQ, MCQ-30 and neuropsychological test scores, and then, we performed a subtraction between MMQ scores and adjusted cognitive scores. Because MMQ scale is mainly focused on memory issues, we calculated the indices with the two episodic memory tests (Free and Cued Selective Reminding Test and Rey-Osterrieth Complex Figure-memory at 3 min) and with the mean global cognitive performance.

### Statistical analysis

Statistical analyses were performed using SPSS Statistics 26.0. Because of the sample size and the absence of normality distribution, we used non-parametric tests. We used the Mann–Whitney U test for mean comparisons between two groups (PCC vs. HC) and Kruskal-Wallis for comparison between three groups (PCC-CP, PCC-CI, HC). Spearman correlation scores were used to assess the correlation between metacognition scores, and fatigue and neuropsychiatric scales. Effect sizes were estimated with rank biserial correlation and eta squared. A *p*-value <0.05 was considered statistically significant. To control for multiple comparisons, a false discovery rate (FDR) correction was applied.

## Results

### Demographic and clinical characteristics

Demographic and premorbid characteristics were compared between the PCC and HC groups. No significant differences were found in age, sex or years of education. Premorbid risk factors such as hypertension, diabetes and dyslipidemia showed no significant differences. Only tobacco smoking was more prevalent in the PCC group compared to HC (Table 1).

**Table 1:** Demographic and premorbid risk factors of PCC and HC.

		PCC	HC	U/Chi^2^	*p*-value
Demographics	Age	51.45 (8.74)	49.55 (8.84)	0.010	0.244
	Years of evolution education	16.19 (2.82)	16.98 (2.37)	9.763	0.097
	Sex, female %	78.4%	85.7%	1.044	0.307
	Months of evolution	52.12 (5.62)	–	–	–
Premorbid risk factors	Hypertension %	23.0%	16.3%	0.804	0.370
	Diabetes %	9.5%	4.1%	1.257	0.262
	Dyslipidemia %	35.1%	26.5%	1.008	0.315
	Tobacco smoking %	17.6%	2.0%	7.046	**0.008**
	Hospitalization %	29.7%	–	–	–

Values are shown as mean (SD) or *n* (%). Statistically significant *p*-values are shown in bold. PCC, post-COVID condition; HC, Healthy Controls.

Demographic and premorbid risk factors were also compared between the three groups (PCC-CP, PCC-CI and HC). A significant age difference was observed across groups (*p* = 0.002), with the PCC-CP being older (54.28 ± 8.04) than both the PCC-CI (47.52 ± 8.23) and HC (49.55 ± 8.84) groups. There were no differences in sex, years of education, and months of evolution, which was compared only between the two PCC groups. Tobacco smoking remained more prevalent in PCC subgroups (PCC-CI: 19.4%; PCC-CP: 16.3%; HC: 2%; *p* = 0.027). Other premorbid risk factors did not show significant differences (Table 2).

**Table 2:** Demographic and premorbid risk factors of PCC-PC, PCC-CI, and HC.

		PCC-CP	PCC-CI	HC	H/Chi^2^	*p*-value
Demographics	Age	54.28 (8.04)	47.52 (8.23)	49.55 (8.84)	6.555	**0.002** ^**a,c**^
	Years of evolution education	16.28 (2.70)	16.06 (3.02)	16.98 (2.37)	1.356	0.262
	Sex, female %	76.7%	80.6%	85.7%	1.224	0.542
	Months of evolution	52.16 (5.87)	52.06 (5.35)	–	0.757	0.941
Premorbid risk factors	Hypertension %	18.6%	29.0%	16.3%	2.014	0.365
	Diabetes %	9.3%	9.7%	4.1%	1.261	0.532
	Dyslipidemia %	30.2%	41.9%	26.5%	2.147	0.342
	Tobacco smoking %	16.3%	19.4%	2.0%	7.215	**0.027**
	Hospitalization %	34.9%	22.6%	–	1.305	0.253
Neuropsychological assessment	Digit Span Backwards	4.74 (1.25)	3.74 (1.03)	4.71 (1.30)	14.015	**0.001** ^a,b^
	Stroop W	102.67 (18.08)	73.65 (31.294)	107.10 (13.81)	23.157	**<0.001** ^a,b^
	Stroop WC	43.00 (7.86)	28.23 (12.39)	45.19 (8.18)	36.491	**<0.001** ^a,b^
	SDMT	49.02 (10.33)	34.23 (12.13)	55.39 (8.31)	46.836	**<0.001** ^a,b,c^
	FCSRT delayed total recall	14.88 (2.11)	11.87 (4.16)	15.27 (1.28)	27.688	**<0.001** ^a,b^
	ROCF 3’	22.60 (5.87)	20.93 (6.71)	21.86 (5.44)	0.772	0.680
	JLO	24.09 (3.92)	21.32 (5.27)	24.49 (4.11)	8.841	**0.013** ^a,b^
	VOSP (NL)	9.30 (1.03)	8.35 (1.66)	9.22 (1.02)	7.618	**0.022** ^a,b^
	Semantic fluency	23.28 (5.96)	17.97 (5.28)	25.49 (5.35)	26.463	**<0.001** ^a,b^
Global metacognition scales	MMQ-Satisfaction	25.04 (10.76)	20.06 (10.79)	45.53 (12.76)	59.174	**<0.001** ^b,c^
	MMQ-Ability	30.90 (12.11)	23.96 (10.85)	51.91 (8.73)	70.677	**<0.001** ^b,c^
	MMQ-Strategy	43.58 (13.40)	45.93 (12.39)	36.95 (9.89)	12.629	**<0.001** ^b,c^
	MCQ-30	66.25 (17.58)	69.45 (15.57)	58. 57 (12.54)	8.851	**<0.001** ^b,c^

Values are shown as mean (SD) or *n* (%). Statistically significant *p*-values are shown in bold. PCC-CP, post-COVID condition cognitively preserved; PCC-CI, post-COVID condition cognitively impaired; HC, Healthy Controls. Post-hoc analyses revealed statistically significant differences between: ^a^PCC-CP and PCC-CI; ^b^PCC-CI and HC; ^c^PCC-CP and HC.

The PCC-CI group showed higher levels of subjective cognitive symptoms, fatigue, and depression, but there were no differences in anxiety and sleep quality (Table 3).

**Table 3:** Clinical characteristics of PCC-CP and PCC-CI.

		PCC-CP	PCC-CI	U	*p*-value
Clinical characteristics	Fatigue	52.23 (17.88)	65.06 (13.97)	1.770	**0.001**
	Depression	14.00 (7.89)	20.16 (11.41)	5.005	**0.013**
	Anxiety	20.77 (9.77)	24.23 (13.35)	3.744	0.202
	Sleep Quality	10.53 (4.62)	12.52 (5.39)	1.633	0.094
	FLEI-Attention	26.95 (8.27)	32.35 (8.55)	0.181	**0.008**
	FLEI-Memory	29.04 (8.29)	32.03 (6.46)	2.931	0.099
	FLEI-Executive Functions	21.20 (8.88)	28.25 (9.46)	0.849	**0.002**
	FLEI-Total	75.79 (24.11)	90.48 (19.71)	2.295	**0.007**

Values are shown as mean (SD). Statistically significant *p*-values are shown in bold. PCC-CP, post-COVID condition cognitively preserved; PCC-CI, post-COVID condition cognitively impaired.

### Local metacognition

**Figure 1:**
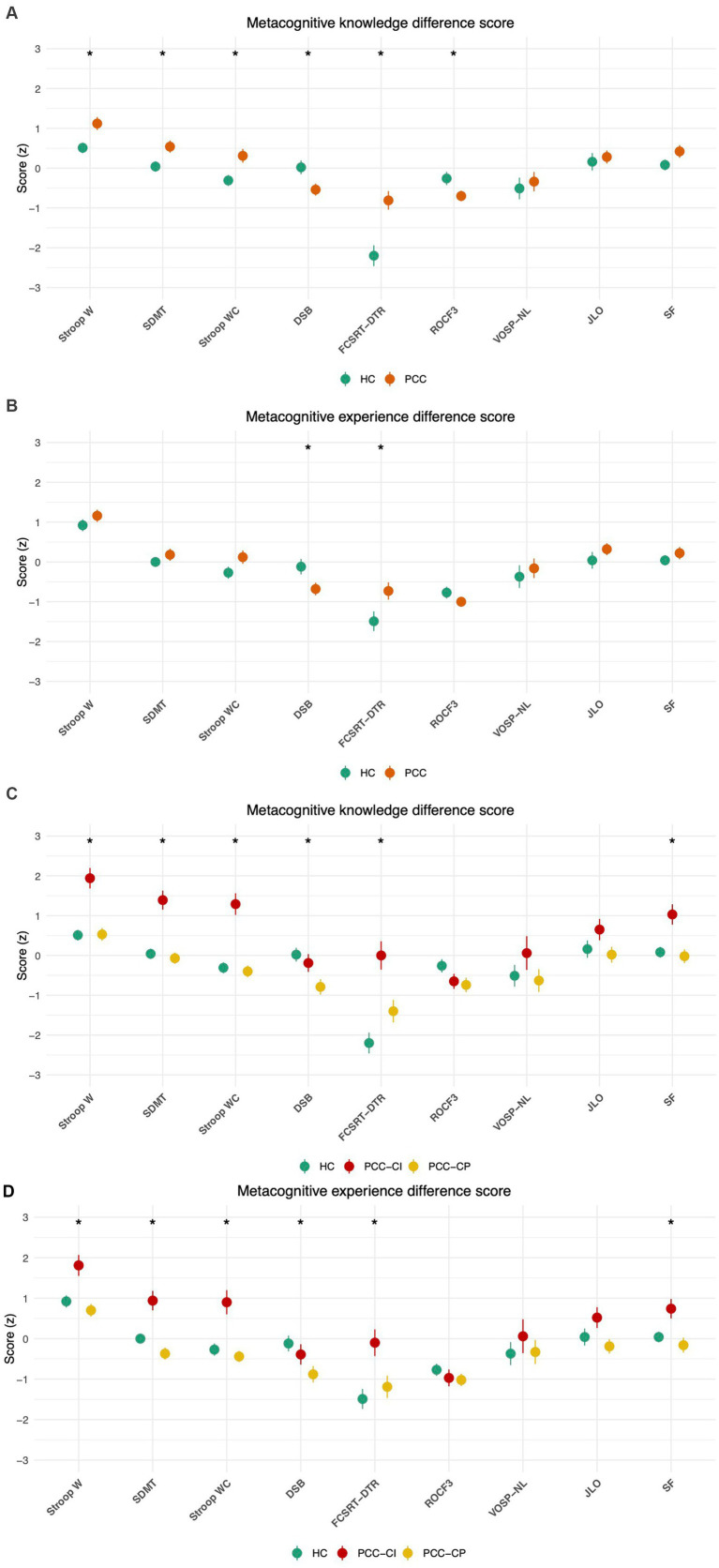
Local metacognition. **(A)** Comparison of metacognitive knowledge in PCC (orange) and HC (green); **(B)** comparison of metacognitive experience in PCC (orange) and HC (green); **(C)** comparison of metacognitive knowledge in PCC-CP (yellow), PCC-CI (red), and HC (green); **(D)** comparison of metacognitive experience in PCC-CP (yellow), PCC-CI (red), and HC (green). Points represent group means; error bars indicate ±1 standard error. Statistically significant differences are shown with an asterisk. Stroop W (word reading); Stroop WC (interference); SDMT, symbol and digits modalities test; DSB, digit span backwards; FCSRT, free and cued selective reminding test (DTR, delayed total recall); ROCF, Rey-Osterrieth complex Figure (3, memory at 3 min); VOSP, visual object space perception battery (NL, number location); JLO, judgment line orientation; SF, semantic fluency; PCC, post-COVID condition; PCC-CP, post-COVID condition cognitively preserved; PCC-CI, post-COVID condition cognitively impaired; HC, healthy controls.

We first compared PCC and HC. PCC scored higher than HC in MKDS for Stroop W, Stroop WC, and in both MKDS and MEDS for Free and Cued Selective Reminding Test delayed total recall. Lower scores were only obtained in PCC in MKDS and MEDS for digit span backwards and in MKDS for Rey-Osterrieth Complex Figure memory at 3 min. These findings are depicted in Figures 1A,B and Table 4, which show that the PCC group was less accurate than the HC group.

**Table 4:** Comparison between PCC and HC in local and global metacognition.

	PCC (*n* = 74)	HC (*n* = 49)	U-Mann Whitney (*p*-value)
*Local metacognition*			
DSB_prediction	−0.54 (1.29)	0.02 (1.21)	1,362 **(0.016)**
DSB_postdiction	−0.68 (1.37)	−0.12 (1.37)	1,387 **(0.024)**
Stroop W_prediction	1.12 (1.40)	0.51 (0.93)	1,307 **(0.007)**
Stroop W_postdiction	1.16 (1.33)	0.92 (1.05)	1,611 (0.280)
Stroop WC_prediction	0.31 (1.48)	−0.31 (0.97)	1,331 **(0.016)**
Stroop WC_postdiction	0.12 (1.43)	−0.27 (1.06)	1,455 (0.082)
SDMT_prediction	0.54 (1.32)	0.04 (0.91)	1,467 (0.062)
SDMT_postdiction	0.18 (1.30)	0.00 (0.84)	1793 (0.914)
FCSRT_DTR_prediction	−0.81 (2.01)	−2.20 (1.84)	1,122 **(<0.001)**
FCSRT_DTR_postdiction	−0.73 (1.88)	−1.49 (1.73)	1,388 **(0.026)**
ROCF3_prediction	−0.70 (1.13)	−0.26 (1.17)	1,360 **(0.035)**
ROCF3_postdiction	−1.00 (1.07)	−0.77 (1.04)	1,530 (0.246)
VOSP_LN_prediction	−0.34 (2.10)	−0.51 (1.91)	1750 (0.743)
VOSP_LN_postdiction	−0.16 (2.12)	−0.37 (2.00)	1,696 (0.540)
JLO_prediction	0.28 (1.41)	0.16 (1.53)	1808 (0.978)
JLO_postdiction	0.32 (1.29)	0.04 (1.48)	1,698 (0.532)
SF_prediction	0.42 (1.36)	0.08 (0.95)	1,555 (0.163)
SF_postdiction	0.22 (1.33)	0.04 (0.91)	1,684 (0.487)
*Global metacognition (MMQ)*			
MMQ-Satisfaction FCSRT_DTR	−1.59 (1.89)	−1.16 (1.74)	1,667 (0.451)
MMQ-Satisfaction ROCF3	−1.80 (1.03)	−0.12 (0.99)	425 **(<0.001)**
MMQ-Satisfaction GCP	−1.27 (0.91)	−0.14 (0.82)	621 **(<0.001)**
MMQ-Ability FCSRT_DTR	−1.91 (2.15)	−1.00 (1.66)	1,437 (0.52)
MMQ-Ability ROCF3	−2.11 (1.33)	0.04 (0.98)	365 **(<0.001)**
MMQ-Ability GCP	−1.58 (1.10)	0.00 (0.77)	419 **(<0.001)**
MMQ-Strategies FCSRT_DTR	0.63 (2.21)	−1.31 (2.10)	942 **(<0.001)**
MMQ-Strategies ROCF3	0.40 (1.52)	−0.22 (1.24)	1,301 **(0.026)**
MMQ-Strategies GCP	0.93 (1.57)	−0.26 (1.17)	971 **(<0.001)**
*Global metacognition (MCQ-30)*			
MCQ-30 DSB	0.91 (1.57)	0.00 (1.39)	1,215 **(0.002)**
MCQ-30 Stroop W	1.60 (1.81)	0.39 (1.40)	1,105 **(<0.001)**
MCQ-30 Stroop WC	1.45 (1.83)	0.22 (1.26)	1,100 **(<0.001)**
MCQ-30 SDMT	1.40 (1.77)	−0.00 (1.25)	929 **(<0.001)**
MCQ-30 FCSRT_DTR	0.70 (2.46)	−1.18 (1.86)	1,003 **(<0.001)**
MCQ-30 ROCF3	0.48 (1.57)	−0.05 (1.25)	1,389 (0.079)
MCQ-30 VOSP_NL	0.26 (2.54)	−0.65 (2.17)	1,440 (0.054)
MCQ-30 JLO	0.99 (1.78)	0.00 (1.63)	1,278 **(0.006)**
MCQ-30 SF	1.21 (1.74)	−0.05 (1.34)	1,036 **(<0.001)**
MCQ-30 GCP	1.00 (1.65)	−0.10 (1.05)	959 **(<0.001)**

Values are shown as mean (SD). Statistically significant *p*-values are shown in bold. Stroop W (word reading); Stroop WC (interference); SDMT, Symbol and Digits Modalities Test; DSB, Digit Span Backwards; FCSRT, Free and Cued Selective Reminding Test (DTR, Delayed Total Recall); ROCF, Rey-Osterrieth Complex Figure (3, memory at 3 min); VOSP, Visual Object Space Perception Battery (NL, number location); JLO, Judgment Line Orientation; SF, Semantic Fluency; PCC, post-COVID condition; HC, Healthy Controls.

When we compared the three groups (i.e., PCC-CI, PCC-CP, and HC), a significant group effect was found in MKDS and MEDS for Stroop W, Symbol Digit Modalities Test, Stroop WC, digit span backwards, Free and Cued Selective Reminding Test-delayed total recall, and semantic fluency. Post-hoc analysis revealed that the PCC-CI group scored higher than both the PCC-CP and HC groups on MKDS and MEDS for Stroop W, Stroop WC, Symbol Digit Modalities Test and Free and Cued Selective Reminding Test-total delayed recall. The PCC-CP group scored lower than the HC group on both MKDS and MEDS backwards digit span. Regarding MKDS for semantic fluency, the PCC-CI group scored higher than the PCC-CP and HC, but no differences were observed between PCC-CP and HC. These results are shown in Table 4 and Figure 1C, which illustrate that PCC-CI patients overestimate their performance compared with PCC-CP and HC before the task, and Figure 1D, which illustrates the overestimation of PCC-CI patients compared to PCC-CP and HC after the completion of the task. That is, in most cognitive domains, PCC-CI patients presented an overestimation of their cognitive performance compared to PPC-CP and HC.

**Figure 2:**
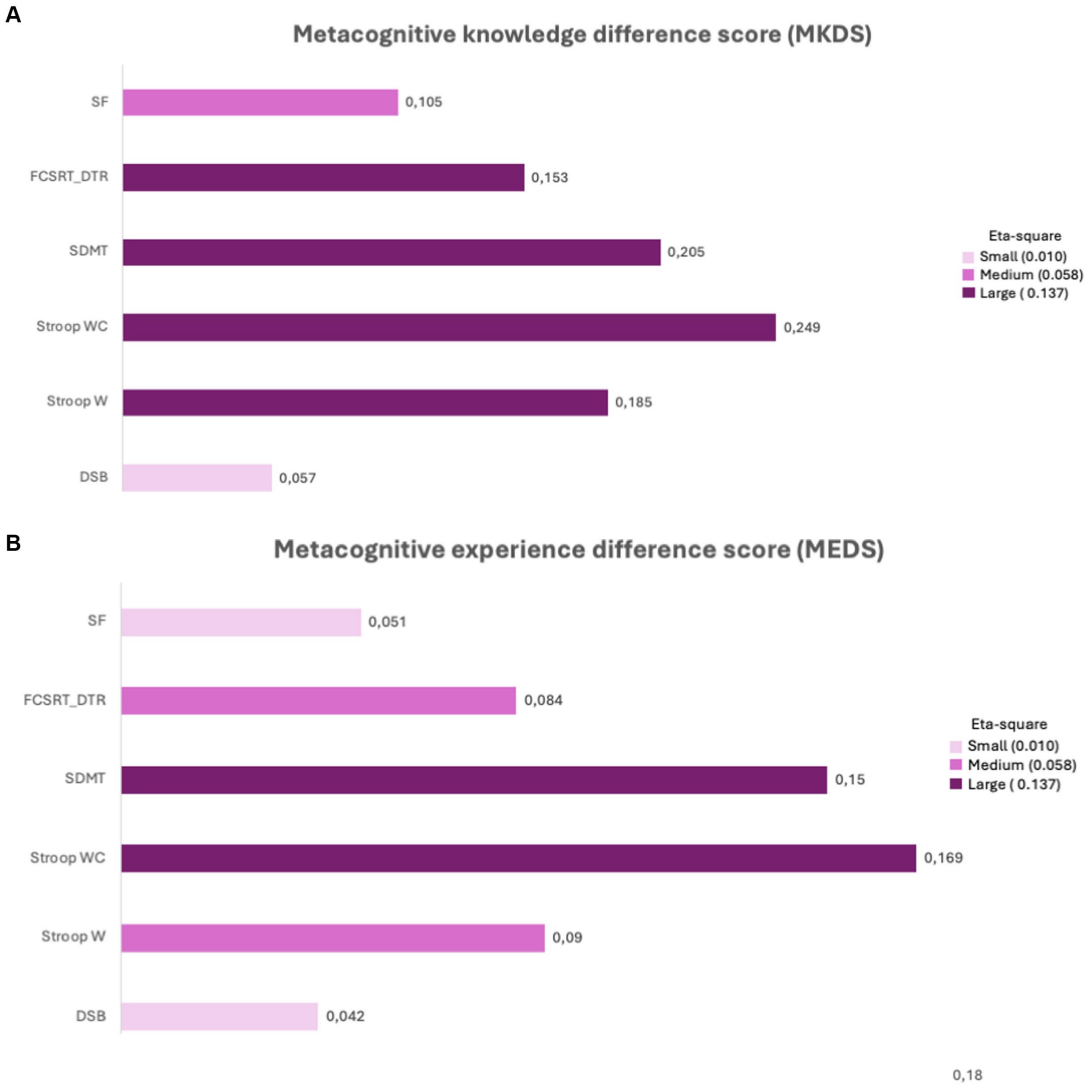
Effect sizes in local metacognition assessments. **(A)** Representation of effect sizes (eta square) for the mean comparisons in metacognitive knowledge between PCC-CP, PCC-CI, and HC; **(B)** Representation of effect sizes (eta square) for the mean comparisons in metacognitive experience between PCC-CP, PCC-CI, and HC. Stroop W (word reading); Stroop WC (interference); SDMT: symbol and digits modalities test; DSB, digit span backwards; FCSRT, free and cued selective reminding test (DTR, delayed total recall); SF, semantic fluency.

In MKDS, effect sizes were large for Stroop W, Stroop WC, Symbol Digit Modalities Test, and Free and Cued Selective Reminding Test-total delayed recall, and medium for semantic fluency. For MEDS, the effect sizes were large for Symbol Digit Modalities Test and Stroop WC, medium for Stroop W and Free and Cued Selective Reminding Test total delayed recall. Effect sizes are depicted in Figure 2A, which shows medium to large effect sizes for group differences in metacognitive knowledge and Figure 2B, which illustrates that the effect sizes for metacognitive experience were also medium to large.

### Global metacognition

**Figure 3:**
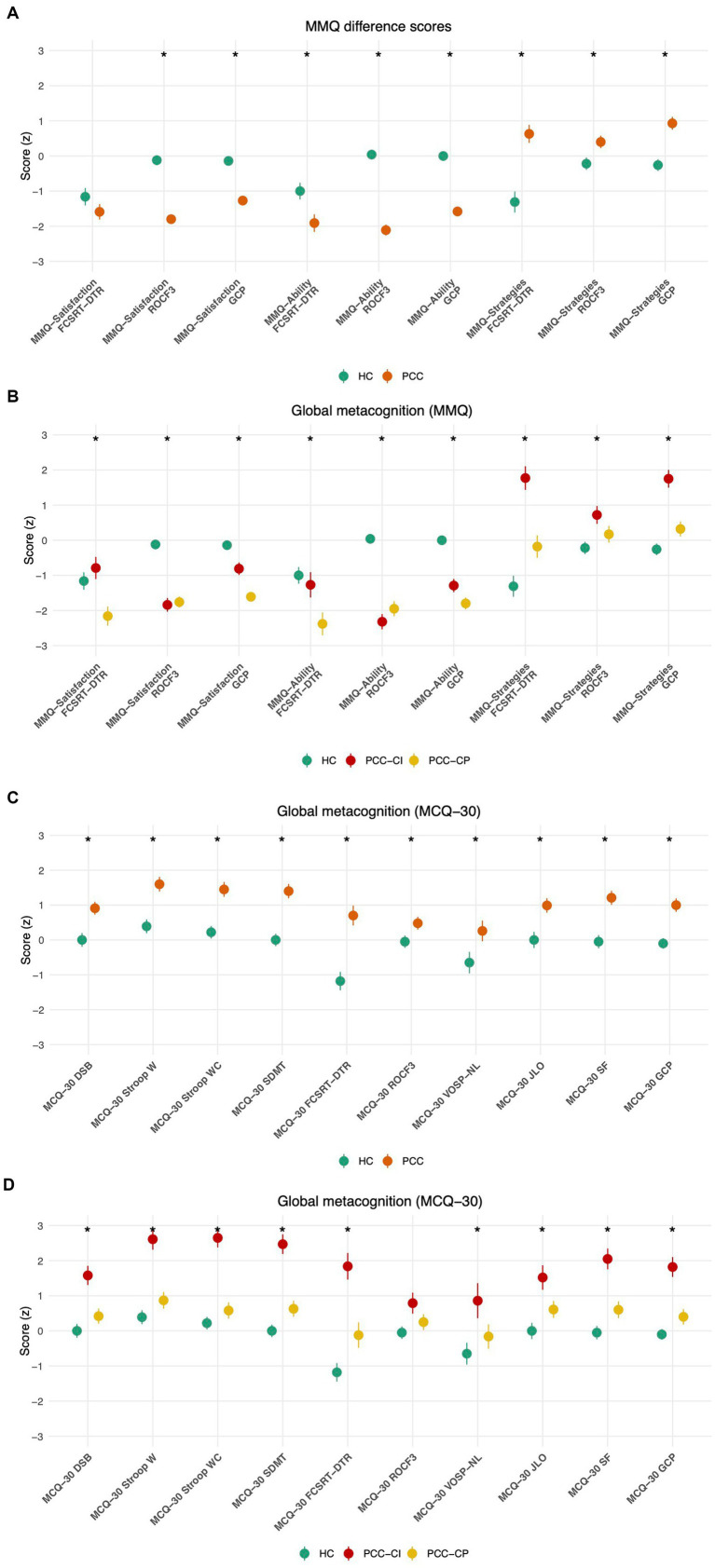
Global metacognition. **(A)** Comparison of global metacognition (MMQ scores) in PCC (orange) and HC (green); **(B)** Comparison of global metacognition (MMQ scores) in PCC-CP (yellow), PCC-CI (red), and HC (green); **(C)** Comparison of global metacognition (MCQ-30) in PCC (orange) and HC (green); **(D)** Comparison of global metacognition (MCQ-30) in PCC-CP (yellow), PCC-CI (red), and HC (green). Points represent group means; error bars indicate ±1 standard error. Statistically significant differences are shown with an asterisk. MMQ-Satisfaction FCSRT_DTR (Multifactorial Memory Questionnaire-Satisfaction, Free and Cued Selective Reminding Test Delayed Total Recall); MMQ-Satisfaction ROCF3 (Multifactorial Memory Questionnaire-Satisfaction Rey-Osterrieth Complex Figure) (memory at 3 min), MMQ-Satisfaction GCP (Multifactorial Memory Questionnaire-Satisfaction, Global Cognitive Performance); MMQ-Ability FCSRT_DTR (Multifactorial Memory Questionnaire-Ability, Free and Cued Selective Reminding Test Delayed Total Recall); MMQ-Ability ROCF3; (Multifactorial Memory Questionnaire-Ability Rey-Osterrieth Complex Figure (memory at 3 min), MMQ-Ability GCP (Multifactorial Memory Questionnaire-Ability, Global Cognitive Performance); Multifactorial Memory Questionnaire-Strategies, (Multifactorial Memory Questionnaire-Strategies, Free and Cued Selective Reminding Test (Delayed Total Recall); MMQ-Strategies ROCF3 (Multifactorial Memory Questionnaire-Strategies Rey-Osterrieth Complex Figure (memory at 3 min); MMQ-Strategies GCP (Multifactorial Memory Questionnaire- Strategies, Global Cognitive Performance); PCC, post-COVID condition; PCC-CP, post-COVID condition cognitively preserved; PCC-CI, post-COVID condition cognitively impaired; HC, Healthy Controls. MCQ-30 Stroop W (Meta-Cognition Questionnaire-30, Stroop W (word reading); MCQ-30 Stroop WC (Meta-Cognition Questionnaire-30, Stroop WC (interference); MCQ-30 SDMT (Meta-Cognition Questionnaire-30, Symbol and Digits Modalities Test); MCQ-30 DSB (Meta-Cognition Questionnaire-30, Digit Span Backwards); MCQ-30 FCSRT (Meta-Cognition Questionnaire-30, Free and Cued Selective Reminding Test) (DTR, Delayed Total Recall); MCQ-30 ROCF3 (Meta-Cognition Questionnaire-30, Rey-Osterrieth Complex Figure (3, memory at 3 min); MCQ-30 VOSP_LN (Meta-Cognition Questionnaire-30, Visual Object Space Perception Battery (NL, number location); JLO, Judgment Line Orientation); MCQ-30 JLO (Meta-Cognition Questionnaire-30, Judgment Line Orientation); MCQ-30 SF (Meta-Cognition Questionnaire-30, Semantic Fluency); PCC-CP, post-COVID condition cognitively preserved; PCC-CI, post-COVID condition cognitively impaired; HC, Healthy Controls.

The PCC group scored lower than the HC group on the metacognitive indices calculated for MMQ-Satisfaction and MMQ-Ability scores in relation to the Rey-Osterrieth Complex Figure at 3 min and global cognition. Furthermore, the PCC group scored higher than the HC group on the MMQ-Strategy in relation to Free and Cued Selective Reminding Test-delayed total recall, Rey-Osterrieth Complex Figure-memory at 3 min, and global cognitive performance (Table 4 and Figure 3A show that PCC patients underestimated their cognitive abilities compared with HC).

Additionally, the PCC group showed higher scores than the HC group on the index derived from MCQ-30, specifically in relation to digit backwards span, Stroop W, Stroop WC, Symbol Digit Modalities Test, Free and Cued Selective Reminding Test-delayed total recall and semantic fluency, Judgment Line Orientation, and global cognitive performance (Figure 3C shows higher scores of PCC patients compared with HC in global metacognition; Table 4).

We also analyzed global metacognition performance across the three groups (PCC-CP, PCC-CI, HC). A significant group effect was observed for the indices based on MMQ-Satisfaction, MMQ-Ability and MMQ-Strategies. Post-hoc analyses revealed that, for MMQ-Satisfaction scores related to Free and Cued Selective Reminding Test-delayed total recall, the PCC-CP group scored lower than the PCC-CI. Regarding MMQ-Satisfaction scores in relation to the Rey-Osterrieth Complex Figure-memory at 3 min, both PCC groups performed lower than the HC group. Similarly, for MMQ-Satisfaction and overall cognitive performance, significant differences were found among all three groups: the PCC-CP group had the lowest scores, followed by the PCC-CI group, while the HC group obtained the highest scores. These findings are summarized in Table 5 and Figure 3B, which shows that both PCC-CP and PCC-CI groups showed lower global metacognition scores than HC.

**Table 5:** Comparison between PCC-CP, PCC-CI and HC in local and global metacognition.

	PCC-CP (*n* = 43)	PCC-CI (*n* = 31)	HC (*n* = 49)	Kruskal–Wallis (*p*-value)	FDR-corrected *p*-value
*Local metacognition*					
DSB_prediction	−0.79 (1.28)	−0.19 (1.25)	0.02 (1.21)	9.009 **(0.011)**	**0.0198**
DSB_postdiction	−0.88 (1.33)	−0.39 (1.40)	−0.12 (1.37)	7.232 **(0.027)**^c^	**0.0405**
Stroop W_prediction	0.53 (1.05)	1.94 (1.43)	0.51 (0.93)	24.671 **(<0.001)**^a,b^	**0.0022**
Stroop W_postdiction	0.70 (1.03)	1.81 (1.44)	0.92 (1.05)	13.097 **(0.001)**^a,b^	**0.0022**
Stroop WC_prediction	−0.40 (0.97)	1.29 (1.51)	−0.31 (0.97)	32.439 **(<0.001)**^a,b^	**0.0022**
Stroop WC_postdiction	−0.44 (0.90)	0.90 (1.66)	−0.27 (1.06)	22.637 **(<0.001)**^a,b^	**0.0022**
SDMT_prediction	−0.07 (0.93)	1.39 (1.33)	0.04 (0.91)	27.028 **(<0.001)**^a,b^	**0.0022**
SDMT_postdiction	−0.37 (0.97)	0.94 (1.34)	0.00 (0.84)	20.377 **(<0.001)**^a,b^	**0.0022**
FCSRT_DTR_prediction	−1.40 (1.85)	0.00 (1.98)	−2.20 (1.84)	20.687 **(<0.001)**^a,b^	**0.0022**
FCSRT_DTR_postdiction	−1.19 (1.81)	−0.10 (1.83)	−1.49 (1.73)	12.307 **(0.002)**^a,b^	**0.004**
ROCF3_prediction	−0.74 (1.19)	−0.65 (1.05)	−0.26 (1.17)	5.118 (0.077)	0.1066
ROCF3_postdiction	−1.02 (1.01)	−0.97 (1.16)	−0.77 (1.04)	1.442 (0.486)	0.5467
VOSP_LN_prediction	−0.63 (1.87)	0.06 (2.36)	−0.51 (1.91)	2.379 (0.304)	0.3648
VOSP_LN_postdiction	−0.33 (1.96)	0.06 (2.33)	−0.37 (2.00)	1.117 (0.572)	0.572
JLO_prediction	0.02 (1.31)	0.65 (1.49)	0.16 (1.53)	4.566 (0.102)	0.1311
JLO_postdiction	−0.19 (1.18)	0.52 (1.43)	0.04 (1.48)	1.239 (0.538)	0.5696
SF_prediction	−0.02 (1.14)	1.03 (1.42)	0.08 (0.95)	14.897 **(0.001)**^a,b^	**0.0022**
SF_postdiction	−0.16 (1.21)	0.74 (1.34)	0.04 (0.91)	8.264 **(0.016)**^a^	**0.0261**
*Global metacognition (MMQ)*					
MMQ-Satisfaction FCSRT_DTR	−2.16 (1.79)	−0.79 (1.75)	−1.16 (1.74)	11.826 **(0.003)**^a^	**0.0032**
MMQ-Satisfaction ROCF3	−1.76 (1.01)	−1.84 (1.08)	−0.12 (0.99)	48.236 **(<0.001)**^b,c^	**0.0015**
MMQ-Satisfaction GCP	−1.61 (0.68)	−0.81 (0.99)	−0.14 (0.82)	43.714 **(<0.001)**^a,b,c^	**0.0015**
MMQ-Ability FCSRT_DTR	−2.38 (2.14)	−1.27 (2.01)	−1.00 (1.66)	10.148 **(0.006)**^a,c^	**0.006**
MMQ-Ability ROCF3	−1.95 (1.41)	−2.32 (1.21)	0.04 (0.98)	54.141 **(<0.001)**^b,c^	**0.0015**
MMQ-Ability GCP	−1.80 (1.11)	−1.29 (1.05)	0.00 (0.77)	49.665 **(<0.001)**^b,c^	**0.0015**
MMQ-Strategies FCSRT_DTR	−0.18 (2.09)	1.77 (1.87)	−1.31 (2.10)	31. 780 **(<0.001)**^a,b^	**0.0015**
MMQ-Strategies ROCF3	0.17 (1.56)	0.72 (1.42)	−0.22 (1.24)	7.081 **(0.029)**^b^	**0.029**
MMQ-Strategies GCP	0.32 (1.40)	1.75 (1.41)	−0.26 (1.17)	30.632 **(<0.001)**^a,b^	**0.0015**
*Global metacognition (MCQ-30)*					
MCQ-30 DSB	0.42 (1.42)	1.58 (1.52)	0.00 (1.39)	18.214 **(<0.001)**^a,b^	**0.0014**
MCQ-30 Stroop W	0.87 (1.56)	2.61 (1.66)	0.39 (1.40)	30.354 **(<0.001)**^a,b^	**0.0014**
MCQ-30 Stroop WC	0.58 (1.53)	2.65 (1.52)	0.22 (1.26)	37.347 **(<0.001)**^a,b^	**0.0014**
MCQ-30 SDMT	0.63 (1.51)	2.47 (1.56)	−0.00 (1.25)	40.347 **(<0.001)**^a,b^	**0.0014**
MCQ-30 FCSRT_DTR	−0.12 (2.38)	1.84 (2.11)	−1.18 (1.86)	28.625 **(<0.001)**^a,b^	**0.0014**
MCQ-30 ROCF3	0.25 (1.48)	0.79 (1.66)	−0.05 (1.25)	4.708 (0.095)	0.095
MCQ-30 VOSP_NL	−0.16 (2.29)	0.86 (2.78)	−0.65 (2.17)	6.024 **(0.049**)^b^	0.054
MCQ-30 JLO	0.61 (1.58)	1.52 (1.94)	0.00 (1.63)	10.625 **(0.005)**^b^	**0.006**
MCQ-30 SF	0.60 (1.57)	2.05 (1.64)	−0.05 (1.34)	26.826 **(<0.001)**^a,b^	**0.0014**
MCQ-30 GCP	0.40 (1.44)	1.82 (1.58)	−0.10 (1.05)	27.849 **(<0.001)**^a,b^	**0.0014**

Values are shown as mean (SD). Statistically significant *p*-values are shown in bold. Stroop W (word reading); Stroop WC (interference); SDMT, Symbol and Digits Modalities Test; DSB, Digit Span Backwards; FCSRT, Free and Cued Selective Reminding Test (DTR, Delayed Total Recall); ROCF, Rey-Osterrieth Complex Figure (3, memory at 3 min); VOSP, Visual Object Space Perception Battery (NL, number location); JLO, Judgment Line Orientation; SF, Semantic Fluency; PCC, post-COVID condition; HC, Healthy Controls. Post-hoc analyses revealed statistically significant differences between, ^a^PCC-CP and PCC-CI; ^b^PCC-CI and HC; ^c^PCC-CP and HC.

For MMQ-Ability scores, post-hoc analysis revealed that both PCC groups scored lower than HC in relation to the Rey-Osterrieth Complex Figure-memory at 3 min and global cognitive performance. Table 5 and Figure 3B show that both PCC-CP and PCC-CI groups obtained lower global metacognition scores than HC.

In contrast, for MMQ-Strategies scores, the PCC-CI group scored higher than PCC-CP and HC in relation to FCSRT total delayed recall. Likewise, for global cognitive performance, PCC-CI scored higher than both PCC-CP and HC groups (Figure 3B; Table 5).

Regarding MCQ-30, the PCC-CI group scored higher than both the PCC-PC and HC group on backwards digit span, Stroop W, Stroop WC, Symbol Digit Modalities Test, Free and Cued Selective Reminding Test-delayed total recall, semantic fluency, and global cognitive performance (Figure 3D; Table 5). Conversely, there were no differences between PCC-CP and HC. All results are shown in Tables 4 and 5.

**Figure 4:**
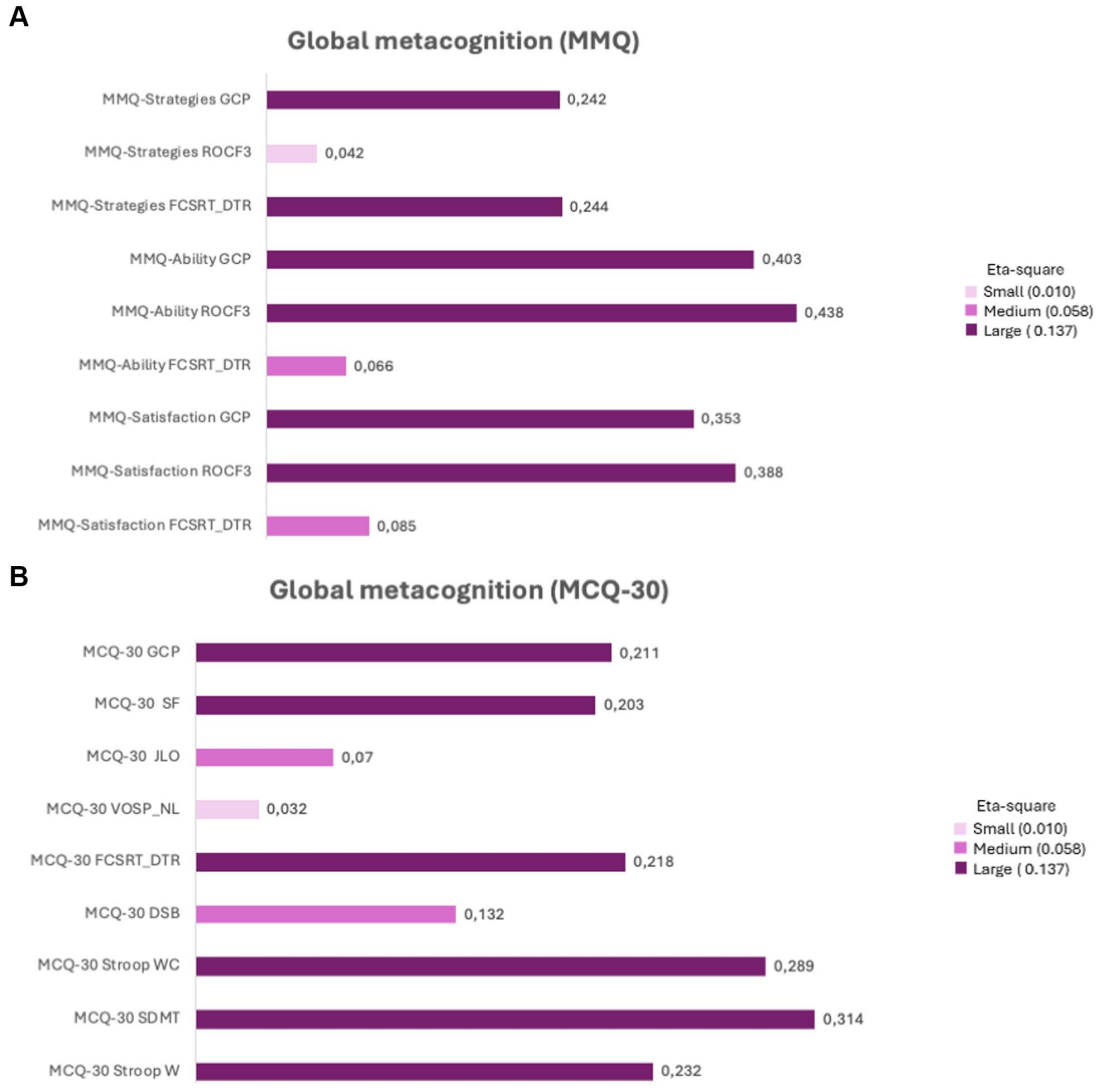
**(A)** Representation of effect sizes (eta square) for the mean comparisons in global metacognition (MMQ scores) between PCC-CP, PCC-CI and HC; **(B)** Representation of effect sizes (eta square) for the mean comparisons in global metacognition (MCQ-30 scores) between PCC-CP, PCC-CI and HC. MMQ-Satisfaction FCSRT_DTR (Multifactorial Memory Questionnaire- Satisfaction, Free and Cued Selective Reminding Test (Delayed Total Recall); MMQ-Satisfaction ROCF3 [Multifactorial Memory Questionnaire-Satisfaction Rey-Osterrieth Complex Figure (memory at 3 min)], MMQ-Satisfaction GCP (Multifactorial Memory Questionnaire-Satisfaction, Global Cognitive Performance); MMQ-Ability FCSRT_DTR (Multifactorial Memory Questionnaire-Ability, Free and Cued Selective Reminding Test Delayed Total Recall); MMQ-Ability ROCF3; (Multifactorial Memory Questionnaire-Ability Rey-Osterrieth Complex Figure (memory at 3 min), MMQ-Ability GCP (Multifactorial Memory Questionnaire-Ability, Global Cognitive Performance); Multifactorial Memory Questionnaire-Strategies, (Multifactorial Memory Questionnaire-Strategies, Free and Cued Selective Reminding Test (Delayed Total Recall); MMQ-Strategies ROCF3 (Multifactorial Memory Questionnaire-Strategies Rey-Osterrieth Complex Figure (memory at 3 min); MMQ-Strategies GCP (Multifactorial Memory Questionnaire- Strategies, Global Cognitive Performance). MCQ-30 Stroop W (Meta-Cognition Questionnaire-30, Stroop W (word reading); MCQ-30 Stroop WC (Meta-Cognition Questionnaire-30, Stroop WC (interference); MCQ-30 SDMT (Meta-Cognition Questionnaire-30, Symbol and Digits Modalities Test); MCQ-30 DSB (Meta-Cognition Questionnaire-30, Digit Span Backwards); MCQ-30 FCSRT (Meta-Cognition Questionnaire-30, Free and Cued Selective Reminding Test (DTR, Delayed Total Recall); MCQ-30 ROCF3 (Meta-Cognition Questionnaire-30, Rey-Osterrieth Complex Figure (3, memory at 3 min); MCQ-30 VOSP_LN (Meta-Cognition Questionnaire-30, Visual Object Space Perception Battery (NL, number location); JLO, Judgment Line Orientation); MCQ-30 JLO (Meta-Cognition Questionnaire-30, Judgment Line Orientation); MCQ-30 SF (Meta-Cognition Questionnaire-30, Semantic Fluency).

Effect sizes were large for almost all the indices and are represented in Figure 4A, (which shows large effect sizes for group differences in global metacognition in MMQ scores: satisfaction, ability, and strategy) and Figure 4B (which shows effect sizes for group differences in MCQ-30 scores).

### Correlations between metacognition indices and other symptoms

Regarding local metacognition, MKDS for Stroop W (*r* = 0.340, *p* = 0.003), Stroop WC (*r* = 0.372, *p* = 0.001) and Symbol Digit Modalities Test (*r* = 0.301, *p* = 0.009) showed positive and moderate correlations with MFIS-Cognitive. No significant correlations were found with the neuropsychiatric scales.

**Figure 5:**
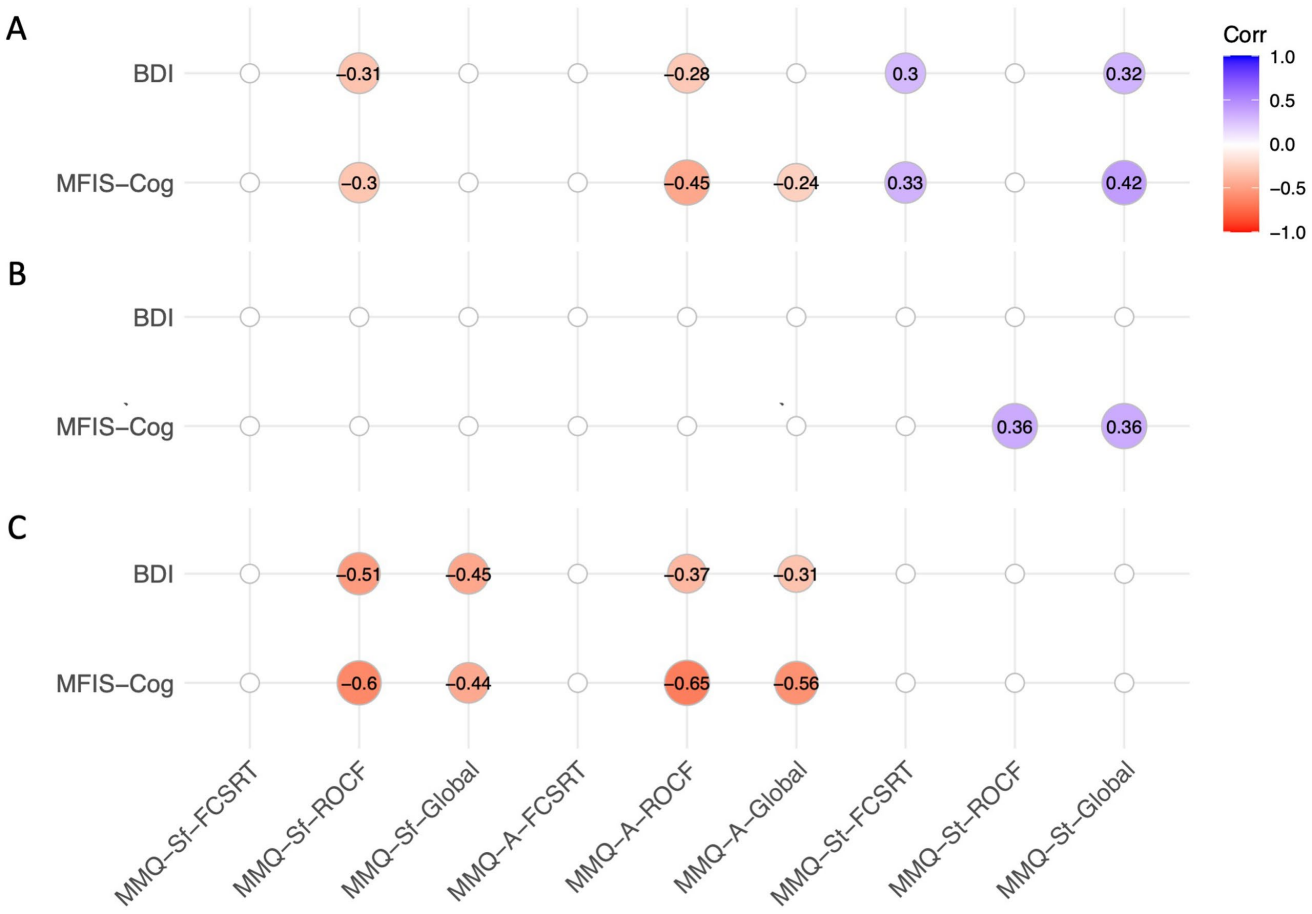
Correlation matrix between global metacognition indices and MFIS-Cog and BDI. **(A)** All PCC patients; **(B)** PCC-CI; **(C)** PCC-CP. Each circle represents the strength and the direction of the correlation (Spearman’s rho). Only statistically significant correlations (*p* < 0.05) are marked.

For global metacognition, we found negative and moderate correlations between MMQ-ability and satisfaction indices with MFIS, and positive and moderate correlations with MMQ-strategies. The PCC-CP group showed negative and moderate correlations between global metacognition indices and fatigue and depression scales, whereas the PCC-CI showed no significant correlations. Correlations are shown in Figure 5.

## Discussion

In this study, we aimed to evaluate local and global metacognitive efficiency in individuals with PCC and the relationship between metacognitive function and clinical symptoms. In general, patients with PCC showed inaccurate judgments in both local and global metacognition compared to HC. Intriguingly, when we classified PCC according to their objective cognitive performance, a different pattern emerged. Patients with PCC-CI overestimated their cognitive performance in local metacognitive tasks mainly in those tasks in which they showed a cognitive deficit, while in global metacognition, both PCC-CP and PCC-CI underestimated their cognitive abilities.

In local metacognition, PCC patients showed less accuracy in assessing their cognitive performance compared to HC, particularly metacognitive knowledge (patient prediction minus actual performance). Specifically, the PCC group showed reduced local metacognitive accuracy in attention, executive function and memory, domains that are commonly impaired in this population (Delgado-Alonso et al., 2022; Voruz et al., 2022; Möller et al., 2023). Similar patterns of domain-specific metacognitive impairment have also been observed in other disorders, where deficits tend to align with the affected cognitive functions (Butzbach et al., 2021). These findings may also suggest that self-monitoring abilities vary across different cognitive domains (McGlynn and Schacter, 1989; Scherling et al., 2016). In contrast, their performance in metacognitive experience (patient postdiction minus actual performance) was inaccurate in only two tests, indicating that PCC patients improve their accuracy after performing the test. This suggests that, although their initial beliefs about cognitive functioning were less accurate, they adjusted their self-evaluations after the assessment (Torres et al., 2016; Duke et al., 2002).

Worth to highlight, patients classified as PCC-CI significantly overestimated their cognitive performance in the domains most commonly impaired in this population, compared to HC. This pattern has been observed in both metacognition knowledge and experience. Such overestimation in PCC-CI patients may reflect the presence of anosognosia, a lack of awareness of one’s impaired function (Kotler-Cope and Camp, 1995), which becomes more prevalent and severe as the disease worsens (Coll-Martinez et al., 2023; Maki et al., 2012; Rosen et al., 2010). Anosognosia has been reported in other disorders associated with structural brain damage (Alzheimer’s disease, multiple sclerosis), even from prodromal stages and generally linked to the severity of cognitive impairment (Coll-Martinez et al., 2023; Bastin et al., 2021; Tagai et al., 2020; Coll-Martinez et al., 2023), but seldom described in PCC patients (Voruz et al., 2022).

Regarding global metacognition, PCC patients (the whole group and also PCC-CP and PCC-CI) showed significantly an underestimation on the MMQ assessment compared to HC. This indicates that they reported more concerns about their memory and a higher use of memory strategies than expected according to their actual cognitive performance compared with HC (Frankenmolen et al., 2017). Notably, PCC-CI patients reported greater use of memory strategies than both HC and PCC-CP, reflecting an attempt to mitigate perceived cognitive difficulties. This underscores the relevance of assessing metamemory to better understand the subjective cognitive complaints which have been associated with reduced quality of life in other conditions (Corti et al., 2025). This discrepancy between PCC patients and HC was also observed and is supported by the results of MCQ-30 (Wells and Cartwright-Hatton, 2004), scoring higher in the PCC-CI group in almost all tests. However, no differences between PCC-CP and HC were observed for the indices calculated for MCQ-30, suggesting that the impairment in global metacognition is less consistent in this group (Wells and Cartwright-Hatton, 2004).

Functional cognitive disorders patients share several symptoms with PCC individuals (e.g., fatigue, cognitive issues, mood change or pain). The typical pattern of metacognitive performance in functional cognitive disorders, characterized by a dissociation between intact local metacognition and impaired global metacognition (Bhome et al., 2022)is however different. The pattern of functional cognitive disorders suggests that, although patients can accurately monitor their cognitive performance while engaged in the tasks, they have difficulty updating their global beliefs about their cognitive functioning based on day-to-day experiences (Teodoro et al., 2023). In contrast, we found that PCC patients presented both local and global metacognition failures. This finding suggests a more widespread alteration in the metacognitive processes, especially prominent in patients displaying objective cognitive deficits. In particular, the overestimation of cognitive capacities observed in PCC with cognitive impairment has been reported in other conditions associated with well-established brain damage (Fowler et al., 2018), but also in healthy population (Dunning-Kruger effect) (Corti et al., 2025).

Studies about neural correlates of metacognition have mainly implicated some regions of the prefrontal cortex, insula, and the parietal association cortex (Vaccaro and Fleming, 2018). Some of these regions have been associated in several neuroimaging studies examining the neural basis of cognitive dysfunction in PCC (Hosp et al., 2024; Díez-Cirarda et al., 2023; Díez-Cirarda et al., 2023; Diez-Cirarda et al., 2025). However, it is worth mentioning that the subgroup of patients with no objective cognitive impairment showed a metacognitive pattern similar to functional cognitive disorders. In this subgroup, global metacognition measures negatively correlated with fatigue and depression (i.e., patients with higher levels of cognitive fatigue underestimated cognitive abilities), confirming the role of these symptoms mediating the relationship between subjective cognitive symptoms and objective cognitive performance (Delgado-Alonso et al., 2023; Denno et al., 2025). It is important to underline that functional cognitive disorder is “a neglected condition, influenced by outdated misperceptions and attitudes, and inadequate knowledge and training” (McLoughlin et al., 2025), and specific studies directly comparing FCD and PCC should be of interest.

An additional factor that should be considered when interpreting our results is about tobacco use. In our sample, smoking was more prevalent in the PCC groups compared to the HC. Previous studies have shown that smoking is associated with a higher risk of developing PCC (Tsampasian et al., 2023). Therefore, smoking could represent a confounding factor contributing to cognitive alterations in PCC patients (Trofor et al., 2024). Future research evaluating the potential role of tobacco consumption, cognitive impairment and neuroimaging in PCC may be of interest.

Our study has some limitations. First, our study was cross-sectional, and metacognition was assessed approximately 4 years after the acute infection. Thus, conclusions about metacognitive abilities in PCC should be restricted to this time frame. Second, we found an underestimation in HC in the index calculated with MMQ-ability and Free and Cued Selective Reminding Test delayed total recall. Although subjective cognitive complaints were an exclusion criterion in the HC group, memory complaints are common in the normal population, which may explain this finding (Potter et al., 2009). However, this observation did not have a major impact on the results because the indices were calculated using several cognitive tests to ensure the consistency of the results. Third, HC were not assessed with the neuropsychiatric and clinical scales. Thus, we cannot exclude that similar associations between metacognition and neuropsychiatric symptoms could also be present in the general population. Fourth, a limitation of this present study is that the MCQ-30 was analyzed using only the total score. Although this approach allowed us to assess global metacognition at a global level (Ramos-Cejudo et al., 2013), it did not capture potential differences across metacognitive subdomains. Future studies with larger sample sizes may benefit from examining the MCQ-30 subscales separately to explore more detailed patterns of metacognitive functioning. Finally, our sample size would be enough to detect medium effect sizes in the main comparisons, but subgroup analyses should be considered exploratory.

In conclusion, this study demonstrates that individuals with PCC exhibit reduced local and global metacognitive accuracy compared to healthy controls. Patients with PCC and objective cognitive impairment overestimated their performance in those cognitive domains commonly affected in this population. In contrast, in global metacognition, PCC patients tend to underestimate their memory abilities and report increased use of memory compensatory strategies. These findings underscore the importance of assessing cognitive performance and metacognitive accuracy in PCC patients to better understand subjective cognitive complaints and inform targeted rehabilitation strategies. Our study paves the way for future studies developing targeted interventions aimed at enhancing metacognitive functioning.

## Data Availability

The raw data supporting the conclusions of this article will be made available by the authors, without undue reservation.
