# Supplementary material for: Metacognition and cognitive dysfunction in post-COVID condition
Source: Front Psychol. 2026 Mar 9;17:1786395. doi: 10.3389/fpsyg.2026.1786395 (PMC13006256; doi:10.3389/fpsyg.2026.1786395)
Supplement: Supplementary file 1 [file Table_1.DOCX]

**SUPPLEMENTARY MATERIAL**

**LEGEND SUPPLEMENTARY FIGURES**

**Supplementary Figure 1. Representation of effect sizes (rank-biserial correlation) for the mean comparisons in metacognitive knowledge between PCC and HC**

**Figure legend:** Stroop W (word reading); Stroop WC (interference); SDMT: Symbol and Digits Modalities Test; DSB: Digit Span Backwards; FCSRT: Free and Cued Selective Reminding Test (DTR: Delayed Total Recall); ROCF: Rey-Osterrieth Complex Figure (3: memory at 3 min); VOSP: Visual Object Space Perception Battery (NL: number location); JLO: Judgment Line Orientation; SF: Semantic Fluency.

**Supplementary Figure 2. Representation of effect sizes (rank-biserial correlation) for the mean comparisons in metacognitive experience between PCC and HC**

**Figure legend:** Stroop W (word reading); Stroop WC (interference); SDMT: Symbol and Digits Modalities Test; DSB: Digit Span Backwards; FCSRT: Free and Cued Selective Reminding Test (DTR: Delayed Total Recall); ROCF: Rey-Osterrieth Complex Figure (3: memory at 3 min); VOSP: Visual Object Space Perception Battery (NL: number location); JLO: Judgment Line Orientation; SF: Semantic Fluency.

**Supplementary Figure 3. Representation of effect sizes (rank-biserial correlation) for the mean comparisons in global metacognition (MMQ scores) between PCC and HC**

**Figure legend:** MMQ-Satisfaction FCSRT_DTR (Multifactorial Memory Questionnaire- Satisfaction, Free and Cued Selective Reminding Test (Delayed Total Recall); MMQ-Satisfaction ROCF3 (Multifactorial Memory Questionnaire-Satisfaction Rey-Osterrieth Complex Figure (memory at 3 min), MMQ-Satisfaction GCP (Multifactorial Memory Questionnaire-Satisfaction, Global Cognitive Performance); MMQ-Ability FCSRT_DTR (Multifactorial Memory Questionnaire-Ability, Free and Cued Selective Reminding Test (Delayed Total Recall); MMQ-Ability ROCF3; (Multifactorial Memory Questionnaire-Ability Rey-Osterrieth Complex Figure (memory at 3 min), MMQ-Ability GCP (Multifactorial Memory Questionnaire-Ability, Global Cognitive Performance); Multifactorial Memory Questionnaire-Strategies, (Multifactorial Memory Questionnaire-Strategies, Free and Cued Selective Reminding Test (Delayed Total Recall); MMQ-Strategies ROCF3 (Multifactorial Memory Questionnaire-Strategies Rey-Osterrieth Complex Figure (memory at 3 min); MMQ-Strategies GCP (Multifactorial Memory Questionnaire- Strategies, Global Cognitive Performance).

**Supplementary Figure 4. Representation of effect sizes (rank-biserial correlation) for the mean comparisons in global metacognition (MCQ-30 scores) between PCC and HC**

**Figure legend:** MCQ-30 Stroop W (Meta-Cognition Questionnaire-30, Stroop W (word reading); MCQ-30 Stroop WC (Meta-Cognition Questionnaire-30, Stroop WC (interference); MCQ-30 SDMT (Meta-Cognition Questionnaire-30, Symbol and Digits Modalities Test); MCQ-30 DSB (Meta-Cognition Questionnaire-30, Digit Span Backwards); MCQ-30 FCSRT (Meta-Cognition Questionnaire-30, Free and Cued Selective Reminding Test (DTR: Delayed Total Recall); MCQ-30 ROCF3 (Meta-Cognition Questionnaire-30, Rey-Osterrieth Complex Figure (3: memory at 3 min); MCQ-30 VOSP_LN (Meta-Cognition Questionnaire-30, Visual Object Space Perception Battery (NL: number location); JLO: Judgment Line Orientation); MCQ-30 JLO (Meta-Cognition Questionnaire-30, Judgment Line Orientation); MCQ-30 SF (Meta-Cognition Questionnaire-30, Semantic Fluency)

**Supplementary Figure 5.** Percentage of impairment of each test in the PCC-CI group, using two different cutoff-scores (scaled-score 7 and scaled-score 5).
