# Supplementary material for: Metacognition and cognitive dysfunction in post-COVID condition
Source: Front Psychol. 2026 Mar 9;17:1786395. doi: 10.3389/fpsyg.2026.1786395 (PMC13006256; doi:10.3389/fpsyg.2026.1786395)
Supplement: Supplementary file 2 [file Data_Sheet_1.PDF]

SUPPLEMENTARY FIGURES

Supplementary Figure 1. Representation of effect sizes (rank-biserial correlation) for the mean comparisons in metacognitive knowledge between PCC and HC

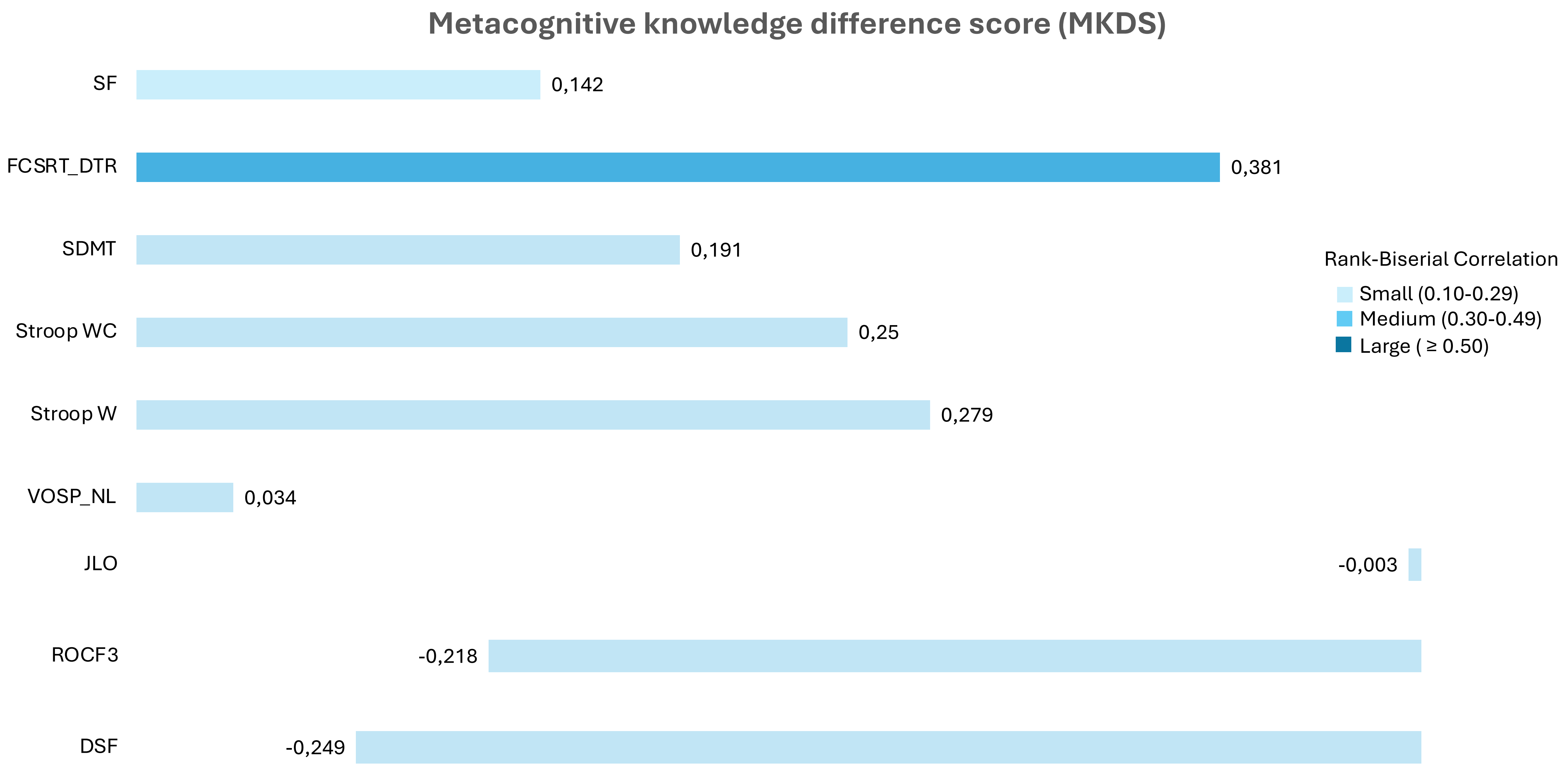

Supplementary Figure 2. Representation of effect sizes (rank-biserial correlation) for the mean comparisons in metacognitive experience between PCC and HC

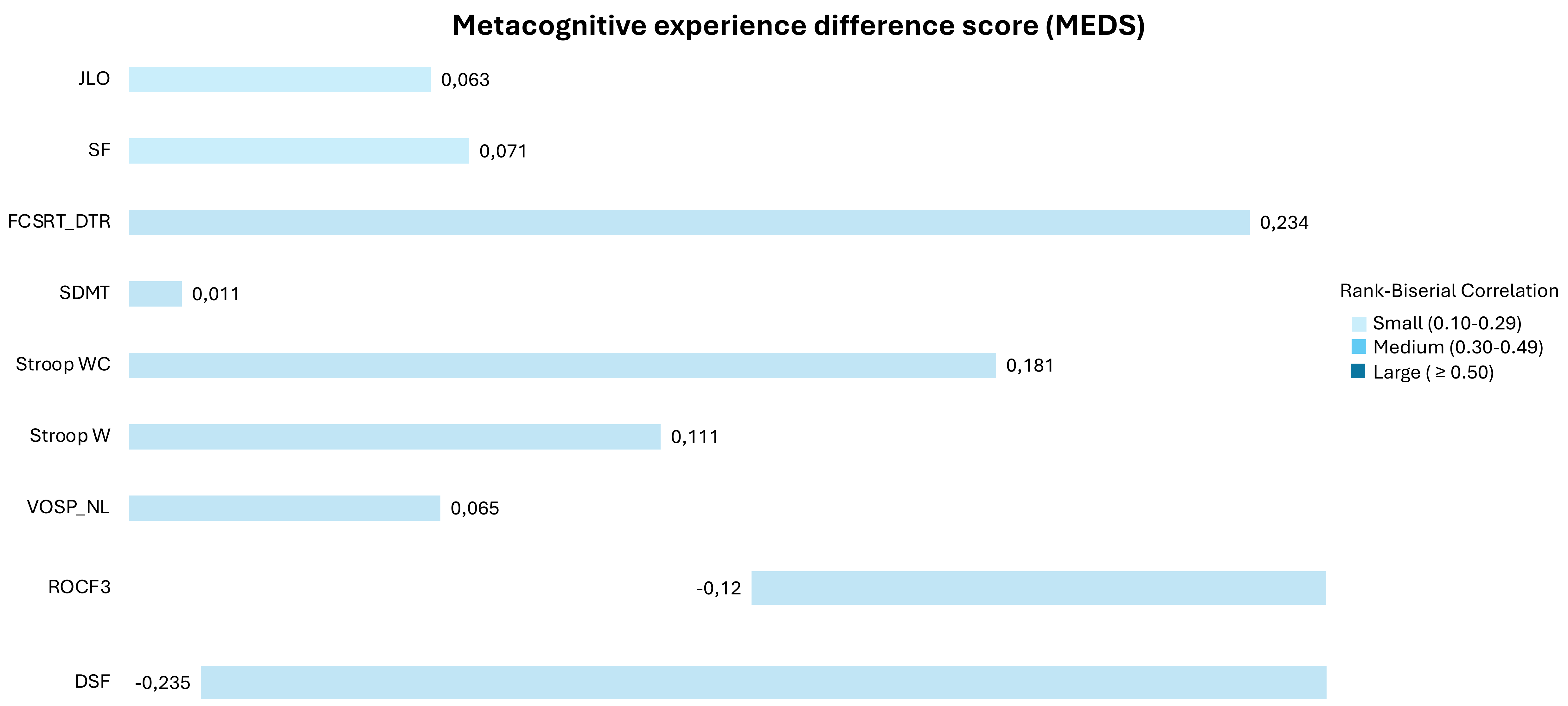

Supplementary Figure 3. Representation of effect sizes (rank-biserial correlation) for the mean comparisons in global metacognition (MMQ scores) between PCC and HC

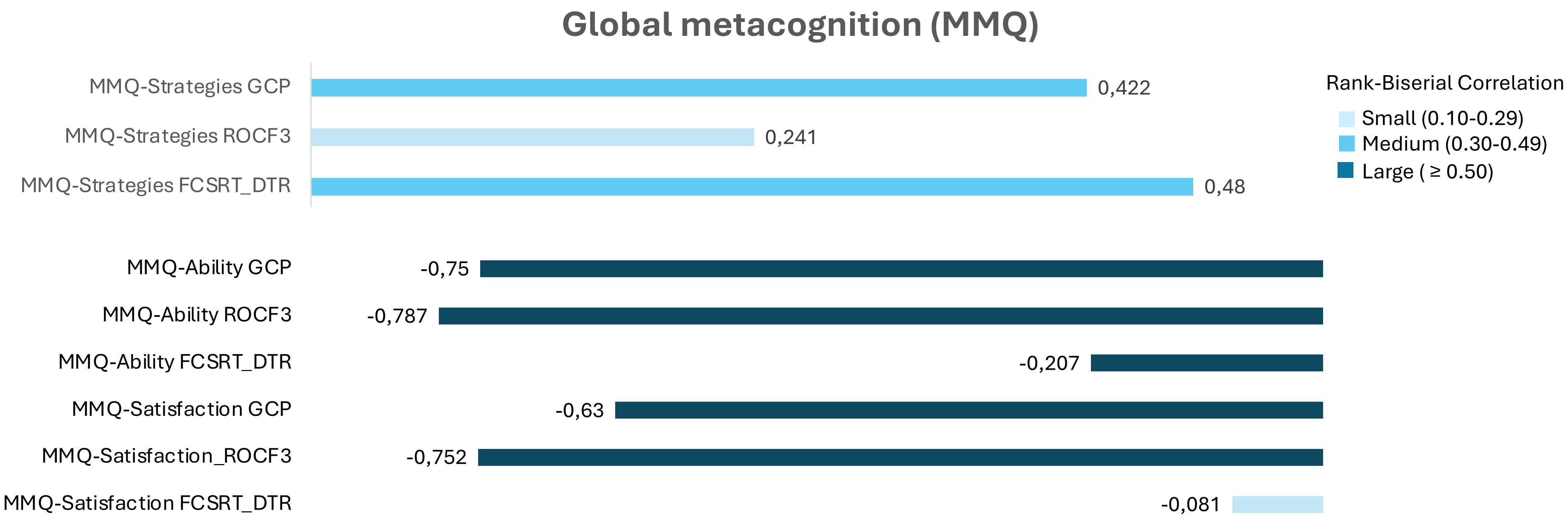

Supplementary Figure 4. Representation of effect sizes (rank-biserial correlation) for the mean comparisons in global metacognition (MCQ-30) between PCC and HC

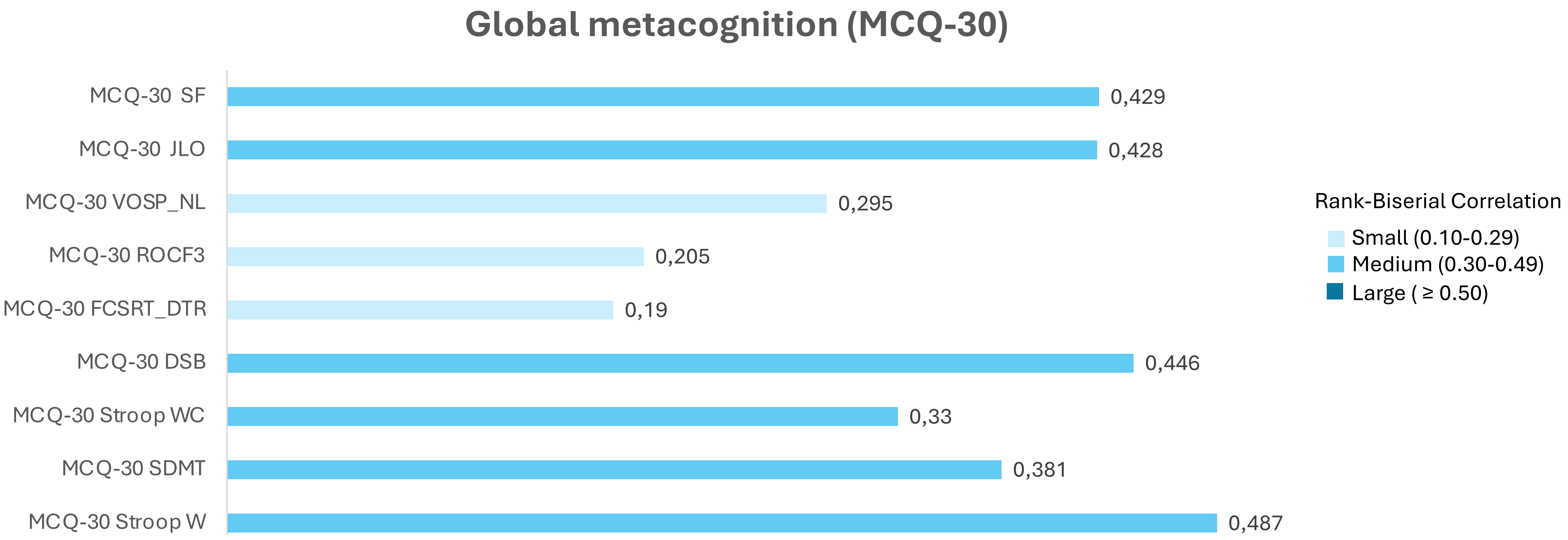

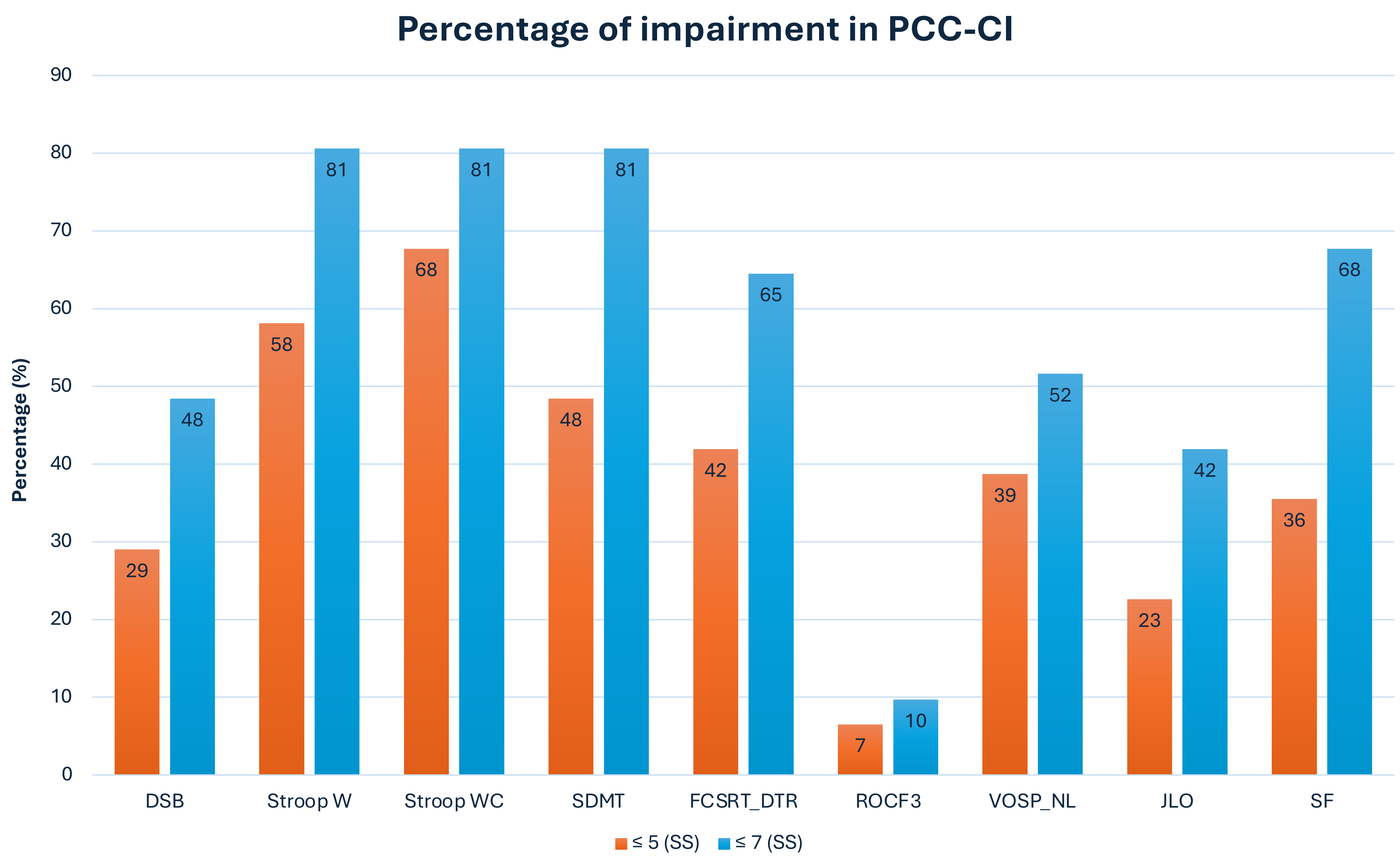
Percentage of impairment in PCC-CI
Percentage (%)
90
80
70
60
50
40
30
20
10
0
29
48
58
81
68
81
48
81
42
65
7
10
39
52
23
42
36
68
DSB
Stroop W
Stroop WC
SDMT
FCSRT_DTR
ROCF3
VOSP_NL
JLO
SF
≤ 5 (SS)
≤ 7 (SS)
